# Moral disengagement and social distancing of people with a personality disorder

**DOI:** 10.1192/j.eurpsy.2021.1177

**Published:** 2021-08-13

**Authors:** R. Heissler, N. Doubková, J. Jonáš, M. Preiss

**Affiliations:** 1 Clinical Research Of Mental Disorders, National Institute of Mental Health, Klecany, Czech Republic; 2 Faculty Of Education, Charles University, Prague, Czech Republic; 3 Faculty Of Arts, Charles University, Prague, Czech Republic; 4 Psychology, University of New York in Prague, Praha, Czech Republic

**Keywords:** social distancing, Moral disengagement, personality disorder, personality functioning

## Abstract

**Introduction:**

People with personality disorders (PD) share some impairments in personality functioning (e.g. identity, intimacy, empathy) that are also associated with inner or interpersonal conflicts, and sometimes also with different strategies of moral disengagement (MD). It is unclear whether MD strategies are related to individuals with/without PD and their willingness to have social contacts with representatives of otherness (like minorities, physically handicapped, etc.).

**Objectives:**

Comparison of the differences in MD strategies and social distance to the otherness of healthy controls and people with PD, and the influence of personality functioning.

**Methods:**

Moral Disengagement Scale which measures eight MD strategies, the Semi-Structured Interview for Personality Functioning DSM-5 assessing the Self and Interpersonal functioning, and Bogardus Social Distance Scale measuring perceived social distance toward various representatives of otherness are applied in two samples (general population and personality disorders).

**Results:**

People with PD showed a significantly higher propensity to use various MD strategies than healthy controls with moderate effect size (.34–.49). Moral disengagement is facilitated by different aspects of personality functioning in both samples, sharing the impairments in maintaining close relationships. Both samples differed in MD strategies connected with higher social distancing.

**Conclusions:**

People with PD are more prone to moral disengagement than healthy adults. MD appears to be facilitated by different aspects of personality functioning in both samples. Some representants of otherness are more related to specific MD strategies. We hypothesize that understanding of specific MD strategies used by people with PD can provide insight and explain some of their behavior.

